# EAgLE: Equivalent Acoustic Level Estimator Proposal

**DOI:** 10.3390/s20030701

**Published:** 2020-01-27

**Authors:** Claudio Guarnaccia

**Affiliations:** Department of Civil Engineering, University of Salerno, I-84084 Fisciano, Italy; cguarnaccia@unisa.it

**Keywords:** noise control, sensor concept, road traffic noise model, dynamic model

## Abstract

Road infrastructures represent a key point in the development of smart cities. In any case, the environmental impact of road traffic should be carefully assessed. Acoustic noise is one of the most important issues to be monitored by means of sound level measurements. When a large measurement campaign is not possible, road traffic noise predictive models (RTNMs) can be used. Standard RTNMs present in literature usually require in input several information about the traffic, such as flows of vehicles, percentage of heavy vehicles, average speed, etc. Many times, the lack of information about this large set of inputs is a limitation to the application of predictive models on a large scale. In this paper, a new methodology, easy to be implemented in a sensor concept, based on video processing and object detection tools, is proposed: the Equivalent Acoustic Level Estimator (EAgLE). The input parameters of EAgLE are detected analyzing video images of the area under study. Once the number of vehicles, the typology (light or heavy vehicle), and the speeds are recorded, the sound power level of each vehicle is computed, according to the EU recommended standard model (CNOSSOS-EU), and the Sound Exposure Level (SEL) of each transit is estimated at the receiver. Finally, summing up the contributions of all the vehicles, the continuous equivalent level, *L_eq_*, on a given time range can be assessed. A preliminary test of the EAgLE technique is proposed in this paper on two sample measurements performed in proximity of an Italian highway. The results will show excellent performances in terms of agreement with the measured *L_eq_* and comparing with other RTNMs. These satisfying results, once confirmed by a larger validation test, will open the way to the development of a dedicated sensor, embedding the EAgLE model, with possible interesting applications in smart cities and road infrastructures monitoring. These sites, in fact, are often equipped (or can be equipped) with a network of monitoring video cameras for safety purposes or for fining/tolling, that, once the model is properly calibrated and validated, can be turned in a large scale network of noise estimators.

## 1. Introduction

The problem of road traffic noise in urban and non-urban areas is becoming more and more important nowadays. The effect of noise on human health is well established [1]. The recent publication of the European Environment Agency (EEA) about “The European environment—state and outlook 2020. Knowledge for transition to a sustainable Europe” [2] lists environmental noise among the most dangerous phenomena, dedicating a full chapter to this issue. In this document, the delay in implementing the actions suggested by the Environmental Noise Directive (END) [3] are claimed, underlining how at least 20% of the EU’s population is still exposed to noise levels unsafe for health. Due to society and human habits, such as to existing infrastructures, road traffic noise is the most important source of noise in the EU, with more than 100 million of people affected by long-term daily average noise levels greater than 55 dBA and with about 80 million of people exposed to night-time levels above 50 dBA [3]. 

In order to cope with this issue, many municipalities introduced fixed or temporary monitoring stations and implemented mitigation actions based on the results of the measurements. Expensive and not always accepted acoustic barriers are the most widespread solution to mitigate the noise produced by the main sources [4]. Pavement plays a key-role in noise emitted by road, as recently studied by many authors aiming to integrate noise reduction with green economy by recycling rubber from old tires into asphalts (rubber asphalts) [5,6,7]. Preventing is also mitigating, thus, innovative solutions, like real time monitoring, are actually studied using a wireless sensor network [8,9].

On the other hand, road infrastructures companies are obliged to perform environmental impact analysis, including noise monitoring and estimation. In addition, when critical situations are highlighted, action plans must be performed, according to the END [2] and to the national regulations of each country. 

In any case, measurements are expensive and cannot be performed all over large areas, thus, road traffic noise predictive models (RTNMs) can be adopted to assess noise produced by vehicles. Extensive reviews of the standard statistical RTNMs can be found for instance in [10,11], also in comparison with field measurements [12]. In [13], a brief review of advanced techniques for road traffic noise assessment is reported, including cellular automata [14], Time Series Analysis [15], Poisson models [16], etc. Can et al. in [17] reported a review of the models to estimate the source power level of the single vehicle.

The usage of advanced computing techniques is somehow growing in literature, even though it must still be demonstrated that the adoption of computationally demanding procedures introduces a widespread benefit in the predictions. In “non-standard” conditions (such as traffic jams or congestions or intersections) usually the common RTNMs fail. Therefore, in large areas case studies, such as big municipalities and big road infrastructures, and for long term average (such as *L_den_* evaluation), the need of a fast and effective model is more important than having an extreme precision (for instance lower than 1 dBA). 

Neglecting the predictive models based on data analysis, such as Time Series Analysis models and Poisson models, it can be affirmed that in order to implement a RTNM it is compulsory to know at least the number of vehicles that pass in a certain time range (flow) and the classification of each vehicle (at least light and heavy categories), together with the geometrical detail of the source and receiver positions. Many other parameters can be included to take into account second order corrections, such as road pavement typology, gradient of the road, temperature, humidity, etc. All these data, compulsory and additional ones, are not always available, and thus the possibility to detect the inputs of any RTNM automatically is an important challenge. In this paper, the author presents the design of a new methodology, the Equivalent Acoustic Level Estimator (EAgLE), based on vehicles detection, counting and tracking, by means of a video processing tool, with a single vehicle noise emission and propagation model. 

There are several studies in literature about image processing, video analysis, object detection and tracking. In [18], a detailed study on vehicle recognition based on deep neural network is presented. Huang in [19] presented traffic speed estimation from surveillance video recordings, highlighting the difficulties related to crowded lanes and perspective corrections. Similar research has been presented by Hua et al. in [20], focusing on the tracking and speed estimation from traffic videos. Biswas et al. in [21] presented a speed estimation performed on video recordings taken by unmanned aerial vehicles. Several other studies are reported in literature, focusing mainly on counting, detection and category assignment, tracking, and speed estimation of vehicles from video recordings. Basically, the real time traffic monitoring systems have been deeply innovated in the last years, leading to the development, and sometimes the installation, of very intelligent sensors on road infrastructures and urban areas. In any case, these sensors are somehow limited, since usually they just detect, count, and track vehicles, in order to help in traffic management, for instance in signalized intersections. Sometimes the video cameras are used to control the restricted areas and, in some cases, also for environmental issues (see, for instance, the Ultra Low Emission Zones (ULEZ) in London), but they usually do not produce an assessment of any environmental parameter, such as air and/or noise pollution. 

The research presented in this paper aims to partially fill this gap, proposing a methodology to embed these sensor networks with a noise level estimator. The proposed approach starts from the recognition of a moving object on the road. Once the object is tracked, it can be counted and categorized according to its dimension. Its speed can be estimated as well, allowing to assess the sound power level. This assessment can be performed in many ways, according to several noise emission models (as presented by Can et al. in [17]). In this paper, the proposed approach is to adopt the CNOSSOS-EU emission model [22]. Once the noise emission, i.e., the source sound power level, of each vehicle is estimated, the overall continuous equivalent level over a given time range can be calculated, summing up all the contributions coming from the vehicles flowing in that time range. This technique, named after “EAgLE: Equivalent Acoustic Level Estimator”, in honor of one of the animals that have the best visual capacities, can be implemented in a new sensor to be developed for road traffic noise assessment purposes. 

The above brief description of the EAgLE methodology is detailed in Section 2, while in Section 3 a preliminary application is presented, showing the results obtained in a preliminary comparison with sound levels recorded on an Italian highway. It will be highlighted that this methodology can be implemented in an existing or under development sensor network. In fact, many road and railway infrastructures, such as many municipalities, have already implemented a video recording network, mainly for safety reasons, that can be easily integrated to become an environmental monitoring network. The integration between existing video recordings and the proposed methodology is the starting point for transforming standard video cameras in smart sensors. In fact, the new proposed sensor, based on video recording, is able to give a quantitative estimation of the noise levels in many points, without the adoption of sound level meters and extensive (and expensive) measurement campaigns. Of course, this methodology being a concept, with only a small dataset for validation, it has several shortcomings at the moment that are reported in the discussion section. The EAgLE efficacy must be tested on a large dataset. This is the reason why a long term validation should be run, for instance, using it in parallel with existing monitoring stations. At any rate, EAgLe seems to be very promising, giving the chance to produce large noise maps, with the only aid of existing, or to be installed, video cameras.

## 2. Materials and Methods

**Figure 1:**
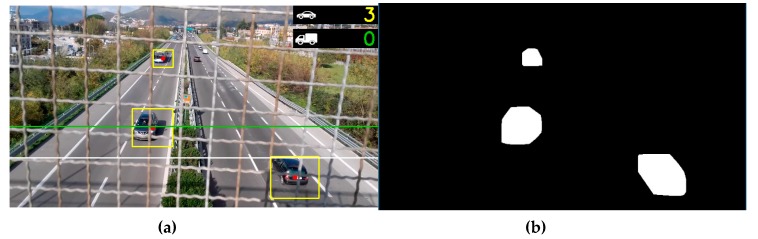
Image analysis on the video frame. (**a**) Moving object are bounded in a yellow box and a red dot (centroid) is applied. Green and white line are used for counting and speed estimation; (**b**) blob detection after background subtraction.

The EAgLE technique adopted in this paper is based on the recognition of any moving object by means of background subtraction, defining a moving “blob” (Figure 1). The blob is bounded in a box (yellow box) and a centroid is applied in the center of the box (red dot). This centroid is tracked and when it passes a given line (green horizontal line in Figure 1), the vehicle is counted and assigned to light or heavy vehicle category according to the box’s diagonal length (see counter on top right of Figure 1). The time each centroid takes for going from the green line to the white line (or vice versa) is used to estimate the speed of the vehicle, after a conversion from frame per second to meter per second. 

The algorithm has been developed in the “Microsoft Visual Studio 2015” framework, with the aid of the “Open Source Computer Vision Library” (OpenCV). The code is written in Python. The source code of the recognition part has been created starting from codes shared in the “GitHub” platform [23]. 

The principal functions of the code are

- *Main* function: it performs calculations needed to count the vehicles and separate the categories. It is also used to estimate the speed of each vehicle;
- *Blob* auxiliary function: it initializes the parameters of the blob, determining the bounding box and diagonal dimensions. In addition, it includes the “*predictNextPosition*” function described below;
- *Header* function: the blob class is defined here, applying the parameters defined in the “blob” function to each continuous mass detected in the frame.

The input file is a MPEG-4 file. At this stage, the algorithm works only in offline mode, analyzing the single frames after having processed the input video. The detection is performed by subtracting the background into two following frames. The tracking of the blob centroid is performed with an improved algorithm, proposed in [23]. The classic approach suggests minimizing the distance between all the centroid positions and the referenced one in two following frames. In this algorithm, a prediction of the position in the next frame is performed for each centroid, on the basis of the trajectory that followed in the previous close frames. Then, a weighted mean between previous positions, with weights varying according to the time distance, is performed and this position is proposed for the following frame. This calculation is done on 4 previous positions, as a compromise between tracking efficiency and computing time. Then, the distance between the predicted position and the real one is minimized, assigning the position to each blob, in all the frames of the video. This is useful to avoid multiple recognition due to several vehicles moving close each other.

When a centroid crosses a chosen line (in our case, the green line in Figure 1), that can be horizontal or vertical, the counting is increased by one. The category is assigned according to the length of the diagonal of the box. A short video sample of this procedure (Video S1) is proposed in the supplementary material of the paper. A time stamp (frame number) of the crossing is recorded and used for evaluation of the speed, by combining this time with the time of crossing the white line. 

Once the vehicle has been detected and classified, and its speed has been assigned to the velocity vector, the sound power level can be estimated. In this preliminary stage, the following procedure has been implemented in Matlab©, but it can be implemented in the same framework of the video processing.

As mentioned in the introduction, among the several emission models that are presented in the literature (see Can et al. [17]), EAgLE implements the CNOSSOS-EU emission model that suggests calculating the sound power level as follows:

(1) $$L_{W,i,m}\left(v_{m}\right)=10log\left(10^{\frac{L_{WR,i,m}\left(v_{m}\right)}{10}}+10^{\frac{L_{WP,i,m}\left(v_{m}\right)}{10}}\right)$$

where, *i* is the index related to the frequency band of octave, *m* is the index related to the type of vehicle, *v_m_* is the average speed of the flow of the *m*-th category of vehicles, *L_w,R,i,m_* is the rolling noise, and *L_w,P,i,m_* is the propulsion noise, given by:

(2) $$L_{WR,i,m}\left(v_{m}\right)=A_{R,i,m}+B_{R,i,m}\;log\left(\frac{v_{m}}{v_{ref}}\right)+\Delta L_{WR,i,m}\left(v_{m}\right)$$

(3) $$L_{WP,i,m}=A_{P,i,m}+B_{P,i,m}\;\left(\frac{v_{m-}v_{ref}}{v_{ref}}\right)+\Delta L_{WP,i,m}\left(v_{m}\right)$$

with *v_ref_* being the reference speed (70 km/h), *A* and *B* table coefficients, and Δ*L_w_* the correction terms. Of course, other emission models can be easily implemented, according to the needs and the country of application of the EAgLE system.

Once the *L_w_* is obtained for each vehicle, the instantaneous sound pressure level at the receiver *L_p_*(*t*) can be estimated using the pointlike source propagation formula, and the single event Sound Exposure Level (SEL) of each pass-by, i.e., the amount of acoustic energy of each transit “compressed” in 1 s, at the fixed receiver, is calculated:

(4) $$SEL=10\log\frac{1}{\;t_{0}\;}\int_{t1}^{t2}10^{\frac{L_{p}\left(t\right)}{10}}\;dt$$

where *t*_0_ = 1 s, *t*_1_, and *t*_2_, respectively, are the beginning and the end of the transit. This step is fundamental in order to make all the transits comparable, since they have strong differences in terms of duration, according to the speed of the vehicles [24]. This procedure is done for each vehicle and for each category, in particular for light and heavy duty vehicles. Then, the overall SEL is calculated with a log sum for light and heavy vehicles. The continuous equivalent level *L_eq_* evaluated in the time range Δ*t* is finally obtained with the following formula:

(5) $$L_{eq}^{\left(\Delta t\right)}=10\log\frac{1}{\Delta t}+10\log\left(\overset{N_{L}}{\underset{i=1}{\sum }}10^{0.1SEL_{i}^{light}}+\overset{N_{H}}{\underset{i=1}{\sum }}10^{0.1SEL_{i}^{heavy}}\right)$$

A résumé of the main steps of the EAgLE methodology is

1. To acquire the video from cameras;
2. To run the counting and recognition algorithm (in real time or in post processing analysis);
3. To remove fake counts and adjust category recognition (only in offline analysis);
4. To feed the noise level estimator with input data;
5. To calculate noise emission levels (according to CNOSSOS-EU);
6. To calculate the SEL of each vehicle;
7. To calculate the overall SEL for light and heavy duty vehicles’ categories;
8. To estimate the *L_eq_* on the required time basis (it should coincide with the video duration).

Of course, once the EAgLE methodology is embedded in existing sensors for video recording and validated with on-site measurements and calibration, the choice of time basis and time range to calculate the *L_eq_* can be tuned according to the needs of the case study. For instance, for urban planning purposes, in urban areas with specific limits, the *L_den_* (i.e., equivalent level evaluated on the day, evening, and night periods, with penalties for evening and night) can be calculated by running the algorithm on the video recordings of one year. Several other applications are possible, changing and tuning the parameters of the EAgLE methodology, depending on the aim of the investigation and on the case study. 

### Preliminary Application on a Case Study on an Italian Highway: Case Study Description

**Figure 2:**
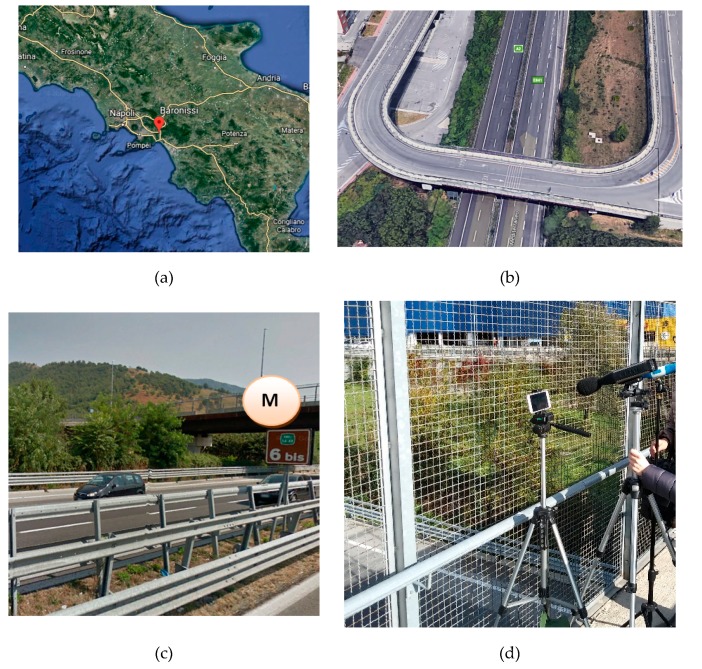
Measurement location: (**a**) Position of Baronissi (red mark), in the Campania region (courtesy of Google Earth©); (**b**) 3D aerial view of the bridge from Google Earth©; (**c**) lateral view of the bridge from Google Street View©; (**d**) picture of the instruments during the measurement collection.

A preliminary application of the EAgLE methodology has been performed on a site located along the Italian highway A2 “Autostrada del Mediterraneo”. This highway is managed by ANAS S.p.a. and goes from the crossing between A30 and RA2, in Fisciano, to Reggio Calabria. The video recording and the measurements have been performed in the city of Baronissi (Figure 2a), in the segment between Fisciano and Salerno, from the sidewalk of a bridge (Figure 2b,c), in safety conditions (Figure 2d). In this segment, the highway is made of two lanes per direction, with an entering lane coming from a gas station, in the south-north direction. Anyway, the entering flow recorded during the measurements was negligible. Furthermore, the traffic on the bridge was negligible. No unusual events have been recorded, such as noisy motorcycles, airplanes passing by, honking, etc., meaning that the conditions of test are quite ideal for the application of the methodology.

The instruments used for the measurements are a class 1 sound level meter Fusion by 01 dB and a video camera embedded in a mobile phone. Two measurements of 15 minutes have been collected around lunch time on Friday, 17 November, 2017. All the acoustic parameters, in particular L_pA,F_, L_eq,A_, percentile levels, acoustic spectrum in third of octaves, etc., and the video of the vehicles passing-by have been recorded in parallel. Temperature was approximately in the range 11 °C–14 °C and wind speed was below 5 m/s on average. Furthermore, to protect the sound level meter from sudden wind peaks, the wind cover was used (see Figure 2d). The flow was running almost freely, with little variations of speed. The average number of vehicles flowing in 15 minutes is 1091 vehicles, with a percentage of heavy vehicles of about 15% in both the measurements. Details about the manual counts performed on the videos are reported in Table 1. 

The detection algorithm is obviously strongly influenced by the stability of the image that is affected by vibrations of the bridge and wind. Since in this sample application a simple camera with a tripod has been used, the overall recognition efficiency is affected by the vibration of the image. Without any post processing and offline analysis, the detection error is greater than 200%. For this reason, in order to check the complete EAgLE technique, a sampling of the two videos was tested, choosing the time ranges in which the camera was more stable, in order to find subsections of the videos less affected by image movements. Two video subsections, each of them made by 5 cuts collected at the beginning, at the end, and in the middle of the video, were extracted, one per each measurement. The overall duration of each subsection is around 300 seconds. Moreover, an offline analysis was run, removing the counts due to the moving of the frames. The periods chosen for the videos’ cuts are summarized in Table 2.

## 3. Preliminary Results

The results of vehicle counting and detection is reported in Table 3, for the two video cuts, approximately five minutes long each, after post processing of the videos and moving frames counts removal.

The efficiency achieved after the removal of moving frames counts is good. Moreover, the recognition is performed with satisfying results. The mistakes in the category are usually overestimated due to the fact that some slightly moving frames could not be removed. That led to the creation of fake moving blobs due to the difference in the background between two following frames. When these fake blobs appeared close to the counting line, they were counted (usually as light vehicles because of the little variation between the two images in the following frames). In addition, it occurred that in some cases two light vehicles moving very closely to each other were recognized as a single heavy vehicle, leading to a small overestimation in this category. The author believes that these problems can be solved by means of a more stable video camera, an optimized angle of view, and a more advanced recognition tool.

**Figure 3:**
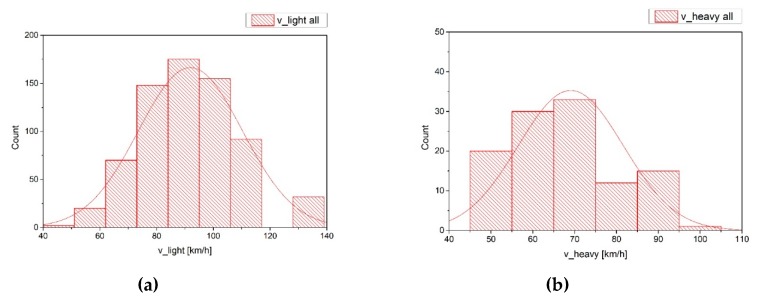
Speeds distributions for light (**a**) and heavy vehicles (**b**) summing the speeds estimated in both the video cuts.

The distributions of the speed estimated with the EAgLE algorithm are reported in Figure 3. It can be noticed that the distribution of light vehicles’ speeds is very close to a normal distribution, as suggested in literature for free flows. For the heavy vehicles, the different shape of the distribution is probably influenced by the mixing of medium and heavy vehicles, which in principle have different average speeds. The EAgLE algorithm run in this preliminary application, in fact, did not distinguish between vans (medium vehicles) and buses or trucks (heavy vehicles). The mean values of the two distributions are of course different, due to the different speed limits and run conditions. 

The missing bins in light vehicles’ speeds distribution figures are due to the discretization in detection of the speed. The frame rate of the camera (30 fps), in fact, influences the speed estimation, that is performed converting the number of frames per second needed to go from the trigger line to the “arrival” line. In particular, the discretization due to the frame rate introduces a discretization in the speed estimated as well. The resulting “delta” is a function of the speed itself (it grows according to the growth of the speed), of the frame rate and of the position of the lines. This position is the result of a compromise between a distance large enough to estimate the speed in a sufficiently large range, and the best location for vehicles pass-by detection. The delta ranges from about 4 km/h in the low speeds part of the distribution to about 14 km/h in the high speeds zone. It is expected that a more advanced camera, with a higher frame rate, will lead to a more precise estimation of the speed, with a consequently better distribution plot. Additional error sources can be the uncertainty on the centroid position, for instance, due to the shadow effect and the resolution of the image, since it influences the bounding box shape. 

Due to the results obtained in the first phase with the detection algorithm, basically, once the identification and the speeds vectors for light and heavy vehicles have been detected, the noise levels estimation has been performed in Matlab framework. As already described in Section 2, the sound power level of the sources has been estimated with the CNOSSOS-EU approach, and the propagation to the receiver has been done with the standard pointlike source propagation formula. The measured continuous equivalent level *L_eq_* on the 15 minutes time range, the levels predicted with some predictive statistical models, the levels predicted with CNOSSOS-EU model, and the *L_eq_* simulated with the EAgLE technique, are resumed in Table 4. The predictive models selected for the comparison are a fully statistical and simple model, i.e., the Burgess model [25], that includes just the traffic flow, the percentage of heavy vehicles, and the distance between source and receiver, and a “semi-dynamical” model, i.e., CNOSSOS-EU, that, in addition to the previous inputs, includes the mean speed of the flow and some correction factors, such as road gradient, temperature, etc.

It can be immediately noticed that the statistical models overestimate the measured *L_eq_*, while the models that consider the speed of the flow (as a mean value, such as CNOSSOS, or for the single vehicle, such as EAgLE) give a much better estimation of the noise levels. 

## 4. Discussion

The preliminary results reported in Section 3 are very encouraging and the comparison performed on the two test videos present a very good agreement between EAgLE simulated levels and the measured *L_eq_*. Furthermore, it should be underlined that at the moment the methodology presents some limitations and shortcomings. 

First of all, the EAgLE technique is strongly affected by the video recording. In particular, the critical points seem to be the angle of recording, which affects the parallax and the conversion between frames and real world distances, the resolution of the camera, which influences the speed estimation, the light conditions, and the shadow effects. The former two points can be quite easily solved with a calibration of the system and with the adoption of high resolution cameras. In regards to the latter two points, of course, a dark image is not feasible for EAgLE at the moment. Problems can occur during the first and last hours of the day, when the sun is barely perpendicular to the road and shadows can modify the size of the bounding boxes, leading to a misclassification of the vehicle. This means that the proposed methodology can be used continuously, during day and night, only in places with artificial lights, but by calibrating the angle of view and the sensibility of the bounding box can include effects of the shadows. It should be also underlined that the actual video recording sensors are always placed on illuminated sites, since it makes no sense to place a video camera on dark sites. For this reason, the EAgLE methodology is still interesting to be embedded in existing sensors, and, for new installation, should be designed in proper locations to avoid the night (or little light) issues and the shadow effects. Moreover, tests with the light projectors of the cars should be performed to see if the recognition efficiency can be kept using the moving lights. Furthermore, tests at different hours of the day have to be performed, to assess the effect of the sunrays inclination on the recognition performance.

Another important issue concerning the video recording is the detection ability in crowded and congested roads. While the exclusion of other “non-noisy” moving objects (pedestrians, animals, etc.) can be performed with a proper placement of the video camera, the possibility of giving bad results in congested situations is a critical point, especially in urban areas. In highways, in fact, congestions are quite rare, especially out of the rush hours. In the author’s opinion, this is a problem that can be solved by improving the detection and classification code of EAgLE. As mentioned in the introduction, several techniques have been developed for this purpose, much more advanced that the one implemented in this preliminary application, based on machine learning, deep learning, neural network, etc. (see for instance [19]). For this reason, the author is confident that great improvements can be done on this issue, by tuning the detection algorithm on the case study under investigation. 

Another limitation of the EAgLE methodology lies in the estimation of non-standard events, such as honking, sirens, extremely noisy vehicles, external sources, etc. It must be underlined that none of the predictive models present in literature nowadays can predict such events, thus, from this point of view, EAgLE, at the moment, is somehow aligned with the other models. In any case, trigger events could be implemented in the recognition code, for instance, using the lights of the ambulances or of the police cars, to tag these events as non-standard and treat them in a proper way.

The preliminary application presented in previous sections is limited due to the small number of measurements and to the free flow condition. More on this part must be done in future researches in order to validate the technique on a larger sample of measurements, with different traffic conditions and geometric features of the sites. 

Looking at the comparison in Table 4, it could be argued that such a strong computation effort is not needed, since the CNOSSOS model gives very similar results. The key point is that the CNOSSOS model needs several inputs to run, while the EAgLE methodology produced excellent results just using the video recordings. Moreover, EAgLE includes a fully dynamic model, since it considers the speed and the kinematics of the single vehicle.

Even with the above mentioned limitations of this study, the EAgLE methodology is really promising, because of its easiness in application in any place controlled by video camera recordings. The actual algorithm is quite easy and can be implemented in real time monitoring, to produce raw estimations of noise levels. Of course, for a more reliable estimation, an offline analysis is mandatory in order to clean the raw data from mistakes in counting or the classification of vehicles, such as in estimating the speed. The integration of this system in a complex sensor, including video recording, online analysis, data transmission, and offline processing, is encouraged by the preliminary results obtained in the case study application. The author believes that with a more powerful video camera network and an improved data processing system, this methodology can be extremely useful in qualitative noise monitoring systems, especially in urban areas and big infrastructures, where usually video recording is already present for safety reasons or for tolling/fining systems.

Future studies should include the production of a test sensor that embeds the EAgLE methodology, with a video camera, a sound level meter, and a processor able to run the algorithm at a local site. In this way, the sensor can be tested on a large scale validation, with a continuous recording of pressure levels and video images in order to test the online performances and the criticisms. A sensibility analysis of the sensor can be performed, testing the variations according to the detection and propagation critical elements (such as angle of view, distance, geometry of the site, etc.) and to the source parameters (such as flow volume, typology and dynamics, pulsing conditions, and/or congestions). Moreover, the non-standard events, such as honking, ambulances, police sirens, etc., should be investigated, since the noise produced is due to both the vehicle and to external loudspeakers. 

Once the EAgLE methodology will be validated, a large spatial scale can be tested, with the aim to produce a noise map of a city or of a transportation infrastructure, taking advantage of the existing video camera networks. When long term recordings are available, for instance, in more than a year, the *L_den_* estimation can be performed, using real traffic data, instead of simulating ideal conditions in noise predictive software. This could help local policy makers and infrastructure managers in finding the critical points of their networks and, if needed, in committing to implement further investigations, based on standard noise level measurements or other tools.

## 5. Conclusions

In this paper, the EAgLE (Equivalent Acoustic Level Estimator) technique has been presented. This technique, based on image analysis, vehicle tracking, and dynamic noise modeling, aims at producing a robust estimation of the continuous equivalent noise level on given time ranges, by using just a video camera recording. 

A preliminary application of the technique, in a short time range (630 s) related to a case study along a highway in South Italy, has been presented, showing how, with a good recognition efficiency, the noise levels estimated with EAgLE are extremely close to the measured levels in this reduced sample of measurements performed in free flow and standard conditions. 

More tests are needed to validate the EAgLE procedure. Moreover, beside the shortcomings discussed in the previous sections, several strength points arise from the first tests. In particular, the possibility to provide reliable qualitative estimations of the noise level in any place embedded with a video camera, in cities, or along transportation infrastructures, is definitively the key point of the proposed sensor. These estimations can be used on one side to cope with the need of a large spatial monitoring, and on the other side to provide first level alarms of exceeding limit thresholds, to be checked with follow-up interventions at specific sites. 

## Figures and Tables

**Table 1:** Details of the manual counts results.

Measurement ID	Starting Time [hh:mm:ss]	Light Vehicles Flow [veh/15 min]	Heavy Vehicles Flow [veh/15 min]	Percentage of Heavy Vehicles [%]
1	12:52:37	930	168	15.3
2	13:16:14	917	167	15.4

**Table 2:** Starting and ending time of the 5 cuts sampled in Video 1 and Video 2.

	Period				
	Period 1 [mm:ss] From–to	Period 2 [mm:ss] From–to	Period 3 [mm:ss] From–to	Period 4 [mm:ss] From–to	Period 5 [mm:ss] From–to
Video 1 cut	00:00–01:02	03:40–04:41	06:03–07:03	10:30–11:30	13:55–15:00
Video 2 cut	00:00–01:18	02:38–03:34	06:29–07:40	10:48–11:51	14:08–15:02

**Table 3:** Results of the manual and Equivalent Acoustic Level Estimator (EAgLE) counting and recognition, after the post processing of the video, in the two video cuts.

	Manual counts		EAgLE Counts		Error Percentage	
	Light Vehicles [counts]	Heavy Vehicles [counts]	Light Vehicles [counts]	Heavy Vehicles [counts]	Light Vehicles [%]	Heavy Vehicles [%]
Video 1 cut (308 s)	334	52	342	55	+2%	+6%
Video 2 cut (322 s)	349	64	355	67	+2%	+5%

**Table 4:** Summary of measured *L_eq_* over 15 minutes compared with predictive model results and with the *L_eq_* simulated with the EAgLE methodology.

Measurement ID	Measured *L_eq_* [dBA]	*L_eq_* Burgess [dBA]	*L_eq_* Cnossos [dBA]	*L_eq_* EAgLE [dBA]
1	75.9	77.9	76.2	76.0
2	75.9	77.9	76.1	75.9

## Supplementary Materials

The following are available online at https://www.mdpi.com/1424-8220/20/3/701/s1, Video S1: 1-minute video of the EAgLE counting algorithm running.
